# Interleukin-17A regulates ependymal cell proliferation and functional recovery after spinal cord injury in mice

**DOI:** 10.1038/s41419-021-04064-1

**Published:** 2021-08-03

**Authors:** Hisao Miyajima, Takahide Itokazu, Shogo Tanabe, Toshihide Yamashita

**Affiliations:** 1 Department of Molecular Neuroscience, Graduate School of Frontier Biosciences, Osaka University, Suita, Japan; 2 Department of Molecular Neuroscience, Graduate School of Medicine, Osaka University, Suita, Japan; 3 Department of Neuro-Medical Science, Graduate School of Medicine, Osaka University, Suita, Japan; 4 Department of Molecular Neuroscience, WPI Immunology Frontier Research Center, Osaka University, Suita, Japan

**Keywords:** Neuroimmunology, Spinal cord injury

## Abstract

Ependymal cells have been suggested to act as neural stem cells and exert beneficial effects after spinal cord injury (SCI). However, the molecular mechanism underlying ependymal cell regulation after SCI remains unknown. To examine the possible effect of IL-17A on ependymal cell proliferation after SCI, we locally administrated IL-17A neutralizing antibody to the injured spinal cord of a contusion SCI mouse model, and revealed that IL-17A neutralization promoted ependymal cell proliferation, which was paralleled by functional recovery and axonal reorganization of both the corticospinal tract and the raphespinal tract. Further, to test whether ependymal cell-specific manipulation of IL-17A signaling is enough to affect the outcomes of SCI, we generated ependymal cell-specific conditional IL-17RA-knockout mice and analyzed their anatomical and functional response to SCI. As a result, conditional knockout of IL-17RA in ependymal cells enhanced both axonal growth and functional recovery, accompanied by an increase in mRNA expression of neurotrophic factors. Thus, Ependymal cells may enhance the regenerative process partially by secreting neurotrophic factors, and IL-17A stimulation negatively regulates this beneficial effect. Molecular manipulation of ependymal cells might be a viable strategy for improving functional recovery.

## Introduction

Spinal cord injury (SCI) is a debilitating neurological condition that can lead to severe and permanent deficits in sensorimotor function. Despite tremendous research efforts, there is still no fully restorable therapeutic strategy for SCI. Regarding the development of therapeutic interventions, one of the main focuses is the modulation of the local environment at the lesion site [1–9]. After SCI, initial damage to the central nervous tissue is followed by many processes such as inflammatory response, degeneration, glial scar formation, and remodeling of the extracellular matrix [1]. Although recent research sheds light on the beneficial aspects of these processes [1, 10], the local environment in the injured spinal cord is inhospitable for neural recovery and regeneration [11–14]. Thus, therapies that can modulate scar formation and provide a growth-permissive environment are awaited.

Ependymal cells are epithelial cells that line the wall of the ventricular system. They are involved in the circulation of cerebrospinal fluid and transportation of substances under physiological conditions [15]. However, it has been reported that ependymal cells can act as endogenous stem cells under pathological conditions such as SCI: ependymal cells are activated after SCI and have strong proliferative ability [2, 4, 6, 16, 17]. Moreover, the ependymal cell-derived scar component has been shown to preserve tissue integrity and provide trophic support for surviving neurons [2, 4, 6, 7]. Thus, targeting ependymal cells to establish a therapeutic strategy to provide a regeneration-permissive local environment seems promising. However, the molecular mechanism underlying ependymal cell regulation after SCI is poorly understood.

Interleukin-17A (IL-17A), an inflammatory cytokine typically produced by immune cells, has been detected in the central nervous system (CNS) under inflammatory conditions. In particular, in multiple sclerosis (MS) and its animal model (experimental autoimmune encephalomyelitis; EAE), IL-17A has been shown to play an important role in disease progression [18, 19]. In addition to its central role as a modulator of the inflammatory response, recent evidence has suggested that IL-17A can modulate neural stem cell (NSC) proliferation both in vitro [20] and in vivo [21]. Moreover, it has been suggested that IL-17A is involved in the pathogenesis of SCI: IL-17A expression is increased in the injured spinal cord [22], and IL-17A deficiency improves locomotor recovery after SCI [23]. However, the detailed role of IL-17A in SCI is still unknown.

In the present study, we investigated the role of IL-17A in ependymal cells after SCI. By using a contusion SCI mouse model, we showed that local inhibition of IL-17A signaling promotes both anatomical and functional recovery, and this treatment enhances ependymal cell proliferation. Furthermore, ependymal cell-specific conditional knockdown of IL-17 receptor A (IL-17RA) was shown to be enough to promote anatomical and functional recovery after SCI. Because the mRNA expression of neurotrophic factors is increased in these mice, it is possible that ependymal cells enhance the regenerative process by secreting neurotrophic factors. Our experiments indicate that molecular manipulation of ependymal cells is a promising strategy for the establishment of new SCI treatments.

## Results

### IL-17A inhibition in the injured spinal cord promotes locomotor recovery after SCI

**Fig. 1:**
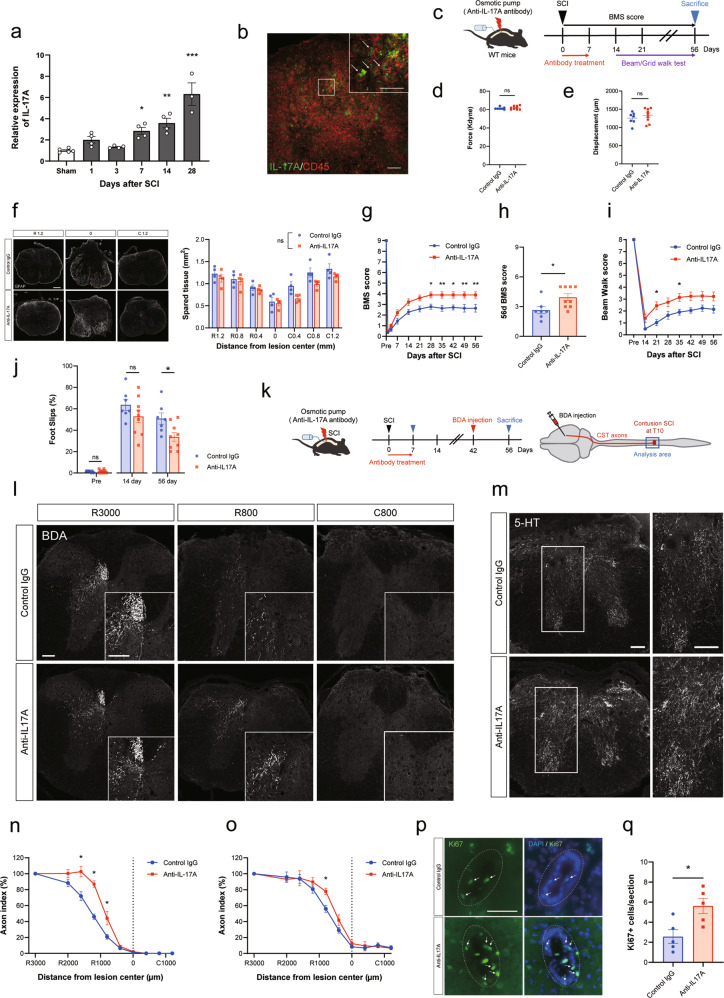
IL-17A neutralizing antibody treatment promotes functional recovery after spinal cord injury (SCI). **a** Relative *IL-17A* mRNA expression in the spinal cord on days 1, 3, 7, 14, and 28 after SCI or sham treatment was measured by real-time polymerase chain reaction. The expression levels in the SCI mice tended to be elevated compared to those in sham-treated mice (*n* = 3–5 mice per time point, *p < 0.05, **p < 0.01, ***p < 0.0001, one-way analysis of variance (ANOVA) with post-hoc Tukey-Kramer test). **b** Representative images of injured spinal cord immunostained for IL-17A (green) and CD45 (red) on day 7 after injury. White arrows indicate cells that co-express IL-17 and CD45. Scale bars: 100 μm (Low magnification) and 50 μm (high magnification). Magnified images are also presented in Supplementary Fig. 1. **c** Experimental time course of IL-17A neutralizing antibody administration and behavioral testing. The antibodies were locally administered through osmotic minipumps for 7 days following SCI, and motor function was evaluated for 56 days thereafter. **d** Impact force (kilodynes) applied to the spinal cord during SCI (Control IgG; *n* = 7, Anti-IL-17A; *n* = 9, *p* = 0.51, Student’s *t* test). **e** Displacement (µm) of the impactor tip upon contact with the spinal cord during SCI (Control IgG; *n* = 7, Anti-IL-17A; *n* = 9, *p* = 0.39, Student’s *t* test). **f** Spinal cord cross-section of the lesion epicenter stained for GFAP at 56 days post injury in IL-17A neutralizing antibody or control IgG-treated mice. Left: The area surrounded by the yellow line indicates spared tissue. Scale bar: 200 μm. Right: quantification of the GFAP positive spared tissue at different distances from the lesion epicenter. There was no significant difference between the groups at any given distance from the lesion epicenter (R rostral, C caudal, two-way ANOVA with post-hoc Bonferroni test, *p* = 0.50). **g**–**j** Basso Mouse Scale score (**g**, **h**), beam walk score (**i**), and grid walk test (**j**) after SCI indicate that the IL-17A neutralizing antibody treatment promotes recovery of motor function (control IgG; *n* = 7, anti-IL-17A; *n* = 9, *p < 0.05, **p < 0.01, two-way repeated-measures ANOVA with post-hoc Bonferroni test (**g**, **i**), and Mann–Whitney *U* test (**h**, **j**)). **k** Experimental time course of antibody administration and biotin dextran amine (BDA) injection. **l** Representative images of BDA-labeled corticospinal tract (CST) axons (white) in the transverse sections of the spinal cord at distances of 3000 µm rostral (R3000), 800 µm rostral (R800), and 800 µm caudal (C800) to the lesion epicenter. Scale bars: 100 μm. **m** Representative images of 5-HT-labeled axons (white) in the transverse sections of the spinal cord at 800 µm rostral to the lesion center. Scale bars: 100 μm. **n** Quantification of the BDA-labeled CST axons at each site. The axon index of the CST was calculated as a ratio to the BDA-positive area 3000 µm rostral to the epicenter (*n* = 4, *p < 0.05, Mann–Whitney *U* test). **o** Quantification of the 5HT-labeled axons. The axon index of the RST was calculated as a ratio to the 5-HT-positive area 3000 µm rostral to the epicenter (*n* = 4, *p < 0.05, Mann–Whitney *U* test). **p** Representative images of immunostaining for Ki67 (green) and DAPI (blue) around the central canal (white broken circles) 7 days after SCI. Scale bar: 50 μm. **q** Quantification of the number of cells double-labeled for Ki67 and DAPI around the central canal. IL-17A neutralizing antibody treatment increased the number of Ki67-positive cells (*n* = 5, *p < 0.05, Student’s *t* test). Data are presented as mean ± SEM. ns no statistical significance.

We first examined temporal changes in the expression levels of IL-17A after SCI. Mice received a moderate/severe contusion injury at the level of 9th–10th thoracic cord, and RNA was extracted from the injured spinal cord at several time points (1, 3, 7, 14, and 28 days post-injury). Using quantitative real-time PCR, it was observed that IL-17A expression increased from the acute phase and continued to increase as time progressed (Fig. 1a). We also performed immunohistochemical analysis and found that IL-17A is mainly expressed by CD45-positive immune cells infiltrating into SCI lesion site (Fig. 1b). Among these cells, IL-17A signal is frequently co-localized with CD68-positive cells (Supplementary Fig. 1).

To investigate the effect of IL-17A inhibition at the lesion site on functional recovery, we locally administered an IL-17A neutralizing antibody or control IgG through an osmotic minipump for 7 days (Fig. 1c). It was confirmed that there were no significant differences in the force or displacement of injury exerted by the infinite horizon (IH) impactor between the group that was administered IL-17A neutralizing antibody or control IgG (Fig. 1d, e). To evaluate tissue sparing, we assessed the lesion volume that was delineated by reactive astrocytes that were positive for GFAP at 56 days after SCI. Quantitative analyses revealed that there was no significant difference in the size of the spared tissues between the two groups (Fig. 1f). We then evaluated hind limb motor function using the BMS test, beam walk test, and grid walk test for up to 8 weeks after SCI (Fig. 1c). We showed that, compared with the control mice, the IL-17A neutralizing antibody-treated mice exhibited improved functional recovery (Fig. 1g–j). These results suggest that IL-17A, which has upregulated expression in the injured spinal cord, has a suppressive role in functional recovery after SCI.

### IL-17A inhibition in the injured spinal cord promotes axonal sprouting and proliferation of cells surrounding the central canal

Having shown that functional recovery was promoted by IL-17A neutralizing antibody treatment, we hypothesized that IL-17A is involved in neural network reorganization after SCI. To address this, we performed an anatomical evaluation of two descending supraspinal pathways: the corticospinal tract (CST) and the raphespinal tract (RST) (Fig. 1k).

For labeling CST axons, we injected BDA, an anterograde tracer, into the hind-limb area of the motor cortex at 6 weeks after SCI. Two weeks later (8 weeks post-SCI), we quantified BDA-positive axons extending from the main CST (Fig. 1l, n). As shown in Fig. 1n, the density of BDA-labeled fibers at the rostral side of the lesion center was significantly increased by IL-17A neutralizing antibody treatment.

Serotonergic axons in the RST are known to modulate motor function, and regenerative growth of these axons after SCI is thought to play an important role in functional recovery [24, 25]. Therefore, we evaluated the outgrowth of serotonergic raphespinal projections, as visualized by immunohistochemistry, with anti-5 hydroxytryptamine (anti-5-HT) antibody at 8 weeks after SCI (Fig. 1m, o). As shown in Fig. 1o, the density of the 5-HT-positive fibers was significantly increased by IL-17A neutralizing antibody treatment.

These results indicate that the local inhibition of IL-17A signaling at the lesion site promotes axonal reorganization of both the CST and the RST, which might be the anatomical basis of improved motor function.

Next, we examined whether IL-17A neutralizing antibody treatment affects SCI-induced reactivity of glial cells by immunohistochemistry. Although there was no clear difference in the number or morphology of astrocytes and microglial cells (Supplementary Fig. 2a–e), the proliferation ability of the cells around the central canal, most likely ependymal cells, was enhanced (Fig. 1p, q). This suggests that IL-17A signaling affects the reactivity of ependymal cells, which are thought to play an important role in the recovery process after SCI [2, 4, 6, 7].

### IL-17A inhibition enhances proliferation of ependymal cells, which express IL-17RA

**Fig. 2:**
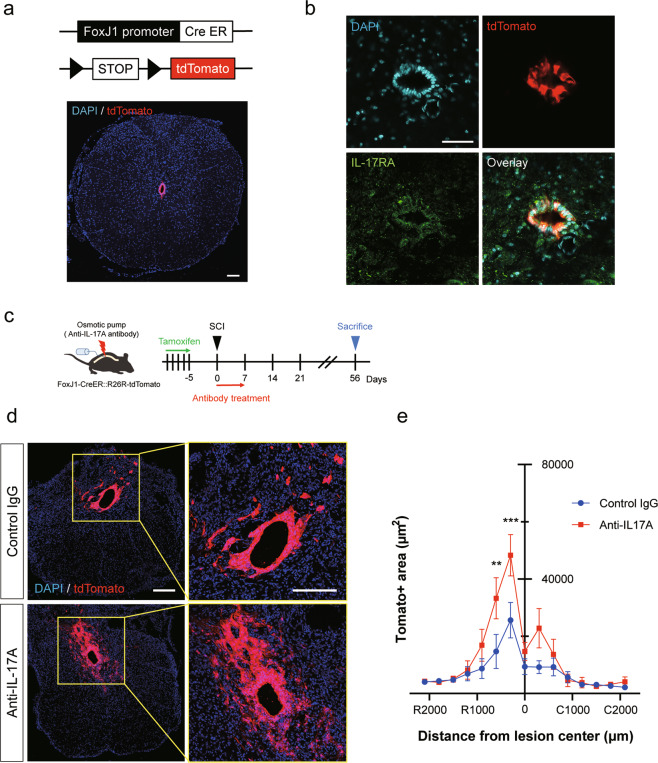
Ependymal cell proliferation is enhanced by anti-IL-17A antibody treatment. **a** FoxJ1-CreER^T2^::R26R-tdTomato mice were used to express tdTomato in central canal ependymal cells in a Cre-dependent manner. Scale bar: 100 μm. **b** Co-immunostaining images of IL-17RA (green), tdTomato (red), and DAPI (blue) in central canal ependymal cells after spinal cord injury (SCI). Scale bar: 50 μm. **c** Experimental time course of tamoxifen and IL-17A neutralizing antibody administration for FoxJ1-CreER^T2^::R26R-tdTomato mice. **d** Representative images of ependymal cell progeny double-labeled for tdTomato (red) and DAPI (blue) in the transversal sections of the spinal cord at a distance of 600 µm rostral from the lesion epicenter 56 days after SCI. Scale bars: 200 μm. **e** Quantification of the tdTomato-positive area. IL-17A neutralizing antibody treatment increased ependymal cell proliferation (control IgG; *n* = 5, anti-IL-17A; *n* = 4, **p < 0.01, ***p < 0.0001, two-way ANOVA with post-hoc Bonferroni test). Data are presented as mean ± SEM.

To confirm the involvement of IL-17A signaling in ependymal cells, we examined the expression of IL-17RA in ependymal cells. We generated a Cre-dependent ependymal cell reporter mouse by crossing R26RtdTomato mice with FoxJ1CreER^T2^ mice, which express CreER^T2^ specifically in ependymal cells [2, 4, 6] (Fig. 2a). We then examined IL-17RA expression in ependymal cells by immunohistochemistry and confirmed that IL-17RA was clearly expressed in ependymal cells (Fig. 2b).

Using FoxJ1CreER^T2^::R26RtdTomato mice, we next examined the proliferation ability of ependymal progeny at the lesion following administration of an IL-17A neutralizing antibody (Fig. 2c). Eight weeks after the SCI, the area of tdTomato-positive ependymal progeny cells seemed to be increased by IL-17A neutralizing antibody treatment (Fig. 2d). We then analyzed the tdTomato-positive area from 2000 μm rostral to 2000 μm caudal to the lesion epicenter and confirmed that the area in the IL-17A neutralizing antibody-treated group was significantly increased compared to that in the control group (Fig. 2e). These results suggest that IL-17A suppresses the proliferation of ependymal cells after SCI.

### Ependymal cell-specific inhibition of IL-17A signaling promotes functional recovery and axonal sprouting

**Fig. 3:**
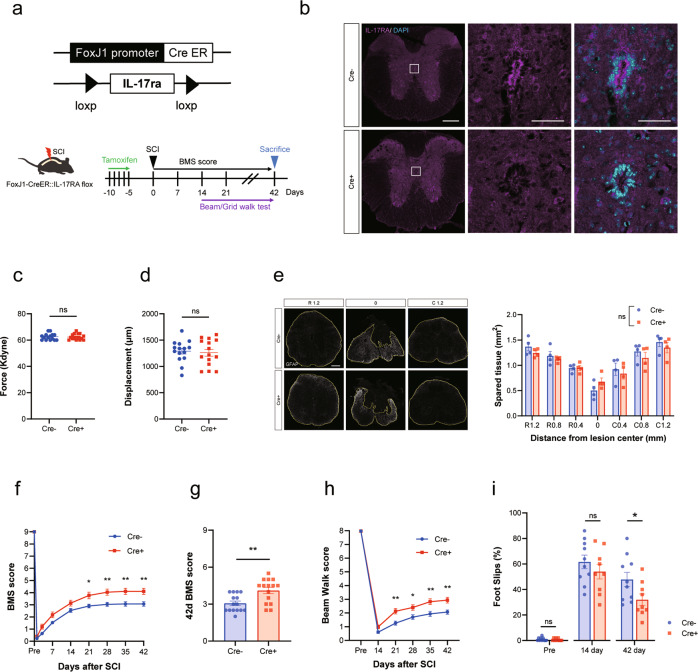
Ependymal cell-specific deletion of IL-17RA promotes functional recovery following spinal cord injury (SCI). **a** Experimental time course of tamoxifen administration and behavioral tests. Cre-dependent ablation of IL-17RA in ependymal cells is achieved by crossing IL-17RA floxed mice and FoxJ1-CreER^T2^ mice. **b** Representative images of ependymal cells immunostained for IL-17RA. Upper: control mouse (Cre- littermate with tamoxifen injection, Lower: IL-17RA-deficient mouse (Cre+ littermate with tamoxifen injection). Scale bars: 200 μm (Low magnification) and 50 μm (High magnification). **c** Impact force (kilodynes) applied to the spinal cord during SCI (*n* = 15 for each, *p* = 0.88, Student’s *t* test). **d** Displacement (µm) of the impactor tip upon contact with the spinal cord during SCI (*n* = 15 for each group, *p* = 0.74, Student’s *t* test). **e** Left: Spinal cord cross-section of the lesion epicenter stained for GFAP at 42 days post injury in IL-17RA-deficient or control mice. The area surrounded by the yellow line indicates spared tissue. Scale bar: 100 μm. Right: GFAP spared tissue area at different distances from the lesion epicenter. There was no significant difference between the groups at any given distance from the lesion epicenter (R: rostral, C: caudal, two-way ANOVA with post-hoc Bonferroni test, *p* = 0.56). **f**–**i** Basso Mouse Scale score (**f**, **g**), beam walk score (**h**), and grid walk test (**i**) after SCI indicate that inhibition of IL-17A signaling in ependymal cells promotes recovery of motor function (**f**–**h**); *n* = 15 for each group, **i**; Cre-: *n* = 10, Cre+: *n* = 9, *p < 0.05, **p < 0.01, two-way repeated-measures ANOVA with post-hoc Bonferroni test (**f**, **h**), and Mann–Whitney *U* test (**g**, **i**)). Data are presented as mean ± SEM. ns no statistical significance.

Although we found that local inhibition of IL-17A at the injury site promoted both ependymal cell proliferation and functional recovery after SCI, an IL-17A neutralizing antibody may affect other cell types in addition to ependymal cells. Therefore, to evaluate the direct effect of IL-17A on ependymal cells under SCI, we generated ependymal cell-specific conditional IL-17RA knockout mice (Fig.3a). We examined the expression of IL-17RA in ependymal cells of this mouse by immunohistochemistry and confirmed that IL-17RA immunoreactivity was effectively reduced (Fig. 3b).

Using this mouse model, we investigated whether IL-17RA deficiency in ependymal cells improved motor function following SCI (Fig. 3a). Based on recent evidence that tamoxifen administration affects the CNS [26], we used Cre- littermates with tamoxifen administration as control mice. The mice received spinal contusion injury similar to that shown in Fig. 1. There was no significant difference between the groups in terms of the intensity or depth of the injury (Fig. 3c, d). We assessed the lesion volume by calculating spared tissue area, and no difference was noted in the size of the spared tissues between the two groups (Fig. 3e).

We evaluated hind limb motor function using the BMS test, beam walk test, and grid walk test for 6 weeks after SCI, and, as expected, ependymal cell-specific deletion of IL-17RA significantly enhanced functional recovery following SCI (Fig. 3f–i).

**Fig. 4:**
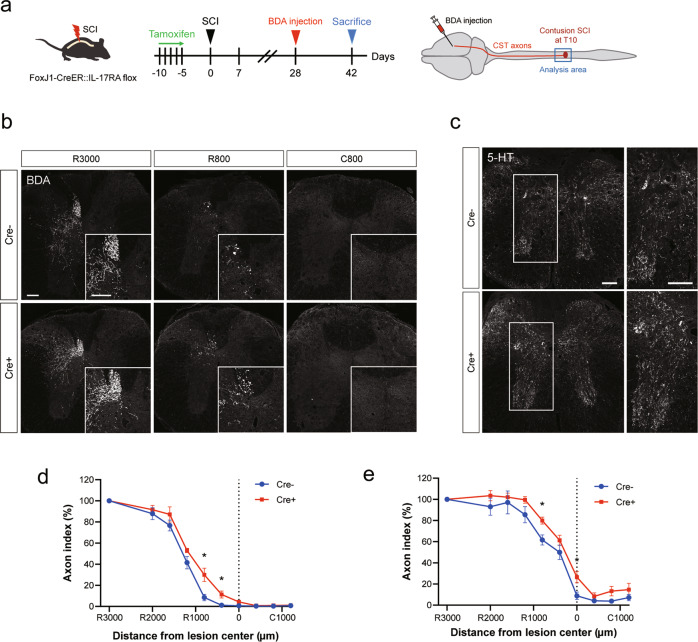
Ependymal cell-specific inhibition of IL-17A signaling enhances axonal sprouting. **a** Experimental time course of tamoxifen administration and BDA injection. **b** Representative images of BDA-labeled CST axons (white) in the transverse sections of the spinal cord at distances of 3000 µm rostral (R3000), 800 µm rostral (R800), and 800 µm caudal (C800) to the lesion epicenter. Upper: Control (Cre-) mouse. Lower: IL-17RA-deficient (Cre+) mouse. Scale bars: 100 μm. **c** Representative images of 5-HT-labeled axons (white) in the transverse sections of the spinal cord at 800 µm rostral to the lesion epicenter. Scale bars: 100 μm. **d** Quantification of the BDA-labeled CST axons at each site. The axon index (BDA-positive area) was increased in IL-17RA-deficient (Cre+) mice (*n* = 5, *p < 0.05, Mann–Whitney *U* test). **e** Quantification of the 5-HT-labeled raphespinal tract axons at each site from the lesion epicenter. The axon index (5-HT-positive area) was increased in Cre+ mice (*n* = 5, *p < 0.05, Mann–Whitney *U* test). Data are presented as mean ± SEM.

We also performed histological analysis of the CST axon (BDA-labeled) and serotonergic RST axon (immunostained for 5-HT) to evaluate the extent of axonal sprouting after SCI (Fig. 4a). Quantification of the proportion of BDA- and 5-HT-positive axons 6 weeks after SCI revealed that axon outgrowth was significantly promoted in the Cre+ mice compared to the control mice (Fig. 4b–e). These results indicate that IL-17A exerts a suppressive effect on functional recovery and axonal reorganization by modulating the ependymal cell response to SCI.

### Inhibition of ependymal cell-specific IL-17A signaling increases neurotrophic factor expression in the injured spinal cord

**Fig. 5:**
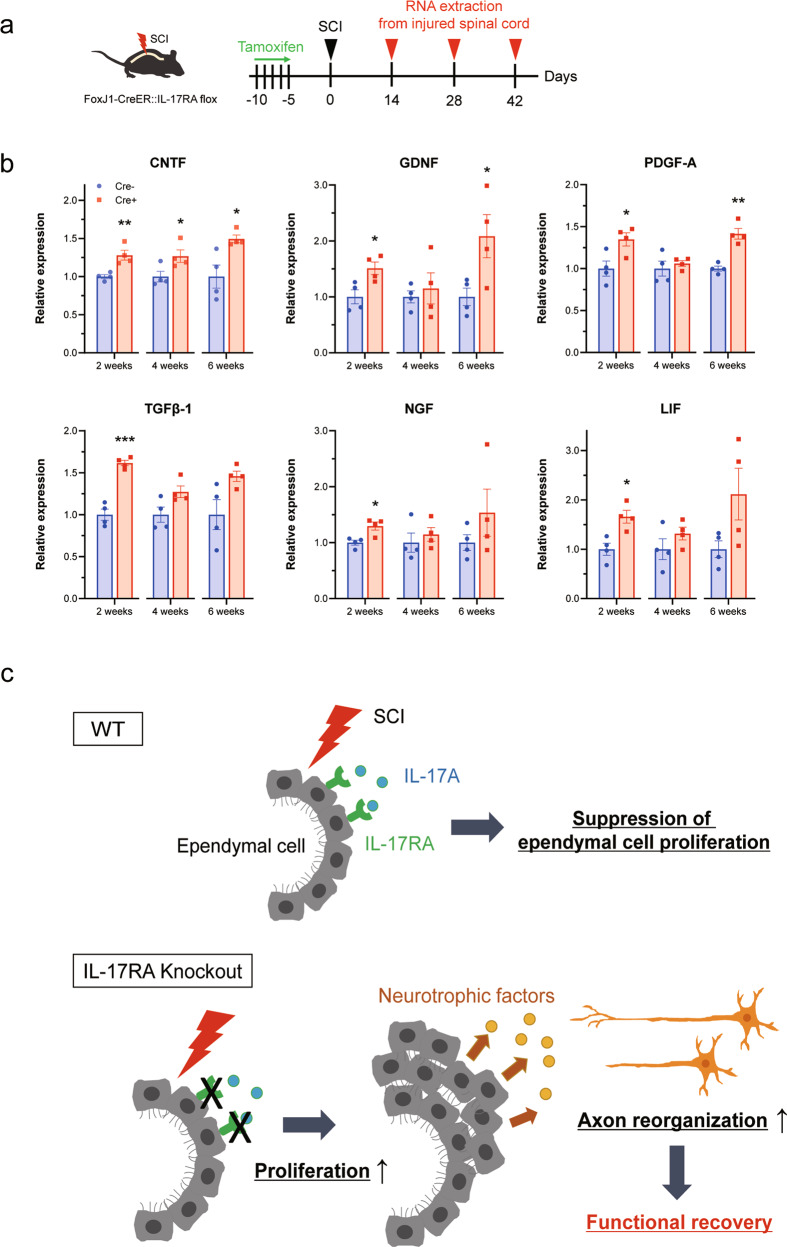
Ependymal cell-specific inhibition of IL-17A signaling upregulates neurotrophic factors in the injured spinal cord. **a** Experimental time course of tamoxifen administration and RNA extraction. **b** Relative mRNA expression of neurotrophic factors in control (Cre-) or IL-17RA-deficient (Cre+) mice spinal cords at 2, 4, and 6 weeks after spinal cord injury (SCI) was measured on real-time polymerase chain reaction. Cre+ mice had significantly higher expression of neurotrophic factors than Cre- mice (*n* = 4 mice per time point, *p < 0.05, **p < 0.01, Student’s *t* test). Data are presented as mean ± SEM. **c** Diagram of the hypothesized mechanism. IL-17A, whose expression level increases in the injured spinal cord, suppresses the proliferation of ependymal cells after SCI. Ependymal cell-specific knockout of IL-17RA promotes ependymal cell proliferation, resulting in increased expression of ependymal cell-derived neurotrophic factors. Then, the reorganization of axons is promoted, resulting in functional recovery.

Neurotrophic factors are one of the main foci of regenerative research after SCI; exogenous delivery of several neurotrophic factors can induce neurite outgrowth or recovery of motor function [8, 27, 28]. SCI is known to trigger the production of neurotrophic factors, and, importantly, ependymal cell progeny is reported to be a major source of neurotrophic support after SCI [6]. Thus, we examined whether ependymal cell-specific inhibition of IL-17A signaling affects neurotrophic factor production in the injured spinal cord by quantifying the mRNAs encoding neurotrophic factors (Fig. 5a). The mRNA expression of neurotrophic factors, including *ciliary neurotrophic factor (CNTF)* and *TGF-β1 (transforming growth factor β1)*, was significantly higher in ependymal cell-specific IL-17RA conditional knockout (Cre+) mice than in control (Cre-) mice (Fig. 5b and Supplementary Fig. 3). These findings suggest that ependymal cells may contribute to the regenerative process after SCI by secreting neurotrophic factors and that IL-17A stimulation negatively influences this beneficial effect (Fig. 5c).

## Discussion

In the present study, we identified IL-17A as a negative regulator of ependymal cell proliferation after SCI. We then demonstrated that ependymal cell-specific conditional knockdown of IL-17RA is sufficient to improve the functional outcome after SCI with enhanced axonal reorganization, providing evidence that manipulation of ependymal cells is a promising therapeutic strategy after SCI.

In the first part of the present study, we confirmed that IL-17A inhibition enhanced functional recovery and axonal reorganization after SCI. Several lines of evidence have suggested that IL-17A is a key component of neuroinflammation [23, 29–31]. Based on the results from preclinical experiments, IL-17A blockade is considered as a potential therapeutic approach for several types of CNS diseases, including MS (EAE) [19], ischemic brain injury [31, 32], and traumatic brain injury [33]. In the case of SCI, increased levels of IL-17A have been reported [22]; however, the therapeutic potential of IL-17A blockade has not been intensively addressed. Hill *et al*. reported that IL-17 knockout mice exhibited improved functional recovery following contusion SCI [23], suggesting that IL-17A signaling has an unfavorable effect on the SCI pathophysiology. Nevertheless, as they used conventional knockout mice, limitations including effects on the development or modification of the systemic immune response should be considered. Therefore, we investigated the effect of local blocking of IL-17A signaling at the lesion epicenter by administering an IL-17A neutralizing antibody using an osmotic pump. As expected, the recovery of motor function was enhanced by this treatment. Importantly, functional recovery was paralleled by enhanced axonal reorganization, in line with the notion that axonal plasticity of the CST and RST could be the anatomical substratum for motor function improvement [24, 25]. Because lesion volume was not affected by IL-17A neutralization, we speculated that the beneficial effect might have been due to a qualitative difference in the scar tissue. Thus, we focused on ependymal cells, which are considered as endogenous NSCs in the spinal cord, and the scar component derived from them were reported to have beneficial functions after SCI [7]. Importantly, IL-17A has been suggested to modulate NSC proliferation and differentiation [20, 21]. Li et al. reported that the IL-17 receptor is expressed on cultured NSCs, and IL-17 stimulation inhibited NSC proliferation without inducing cytotoxicity or apoptosis [20]. Gao et al. also reported the inhibitory effects of IL-17 on the directional differentiation of NSCs, which can be relieved by IL-17 neutralizing antibody treatment [34]. Liu et al. revealed that endogenous IL-17A negatively regulates adult hippocampal neurogenesis [21]. We examined the expression of IL-17RA in ependymal cells, and confirmed that the receptor was densely expressed by them. Based on these observations, we hypothesized that IL-17A negatively regulates ependymal cell proliferation or differentiation under SCI. As expected, our IHC analysis revealed that local treatment of IL-17A neutralizing antibody significantly enhanced ependymal cell proliferation. Further, using FoxJ1CreER^T2^-tdTomato mice to visualize ependymal progeny cells after SCI, we confirmed that the tdTomato-positive area was moderately increased by contusion injury and that it was further increased by IL-17A neutralizing antibody treatment.

Many studies have reported the beneficial properties of ependymal cells after SCI [2, 4, 6, 16, 35]; however, the contribution of ependymal cells is still controversial [36, 37], and the molecular mechanism of their regulation is poorly understood. Previous studies have shown that ependymal cells can proliferate and differentiate into several cell types, including astrocytes and oligodendrocytes, after SCI [2, 4, 6, 35], suggesting that ependymal cells act as NSCs. In addition, the ependymal cell-derived progeny was reported to contribute to axonal elongation and tissue repair by producing neurotrophic factors [6]. However, Ren et al. conducted a fate-mapping study of ependymal cells in different SCI models and claimed that the contribution of ependymal cells to other cell types is minimal; thus, they concluded that ependymal progenitors are not likely to be good candidates for therapeutic targets [37]. In another study, Lacroix et al. addressed the spatiotemporal dynamics of ependymal cell proliferation in both the contusion SCI model and the demyelination model (LPC and EAE) [17]. They observed robust activation of ependymal cells in the SCI model, whereas demyelinating lesions did not trigger ependymal cell proliferation. Therefore, the ependymal cell response to SCI may be different in different experimental injury models, and their contribution to the intrinsic repair capacity is still under debate [17, 35, 37]. Interestingly, marked proliferation of ependymal cells seems to be observed when the depth of the injury is sufficient to reach the CC [4, 17], suggesting that direct physical damage to the CC may trigger ependymal cell proliferation.

Although IL-17A neutralization at the lesion site enhanced both ependymal cell proliferation and functional recovery, which was paralleled by anatomical reorganization, it is highly likely that IL-17A inhibition also affects the inflammatory response via activation or migration of immune cells. In other CNS injury models, including stroke and traumatic brain injury, IL-17A promoted neutrophil infiltration, blood–brain barrier disruption, and the cytotoxicity of CD8+ T cells [38, 39]. Therefore, in order to investigate the ependymal cell-specific contribution, we utilized conditional knockout mice. As a result, our data clearly show that the in vivo manipulation of ependymal cells (ependymal cell-specific conditional knockdown of IL-17RA) is sufficient to improve functional outcomes and enhance axonal reorganization after SCI, supporting the view that this cell population is a promising therapeutic target after CNS injury.

As mentioned above, the progeny of proliferated ependymal cells has been suggested to differentiate into astrocytes and oligodendrocytes after SCI [2, 4]; however, some reports claim that the population of these cells is limited [37], and detailed characteristics and properties of the majority of those cells are not fully understood. Very recently, Llorens-Bobadilla et al. performed single-cell RNA sequencing of fate-mapped ependymal progeny using Foxj1-tdT mice, and showed that the majority of progeny cells expressed the astrocyte marker SOX9 [40]. Moreover, they revealed that ectopic expression of OLIG2 in ependymal cells enhanced ependymal-derived oligodendrogenesis, which contributed to axon remyelination and functional recovery, showing the therapeutic potential of the modulation of resident stem cell fate after SCI [40]. Although we observed a significant amount of ependymal cell-derived scar components following SCI, we did not identify their properties in the present study. Future studies will be needed to define the precise characteristics of ependymal cell-derived progeny under IL-17A signaling inhibition using cutting-edge techniques such as single-cell RNA sequencing. Nonetheless, axonal reorganization and functional recovery were significantly improved by ependymal cell-specific knockout of IL-17 signaling. Because Sabelstrom et al. indicated that ependymal cell progeny is a major source of neurotrophic factors [6], we tested the possibility that manipulation of IL-17 signaling in ependymal cells exerts beneficial effects by producing an increased amount of trophic factors to provide a regeneration-permissive local environment. We examined the mRNA expression of several neurotrophic factors and, as expected, most of them tended to be increased at all time points examined (2, 4, and 6 weeks post-injury). Facilitating neurite outgrowth and neuronal survival by supplying neurotrophins has long been one of the major candidate strategies for SCI treatment, with vast evidence from preclinical investigations [8, 27, 41]. To achieve the continuous administration of neurotrophic factors, direct infusion, viral vectors, biomaterials, and cell transplantation, or a combination of these strategies were tested [8, 27]. However, there are several issues to be solved: non-negligible tissue damage to the continuous infusion site; low stability of trophic factors; formation of inappropriate connections induced by too much trophic factor application [42]; and for cell transplantation therapy, potential teratoma formation, and high cost [43, 44]. Therefore, manipulating resident ependymal cell populations to achieve a regeneration-permissive environment with enhanced neurotrophic support could be a fascinating strategy for the development of new regenerative medicine for SCI. In this regard, our study was the first to identify IL-17A as a regulating factor of ependymal cell proliferation, followed by neurotrophic factor upregulation, providing a concrete example of ependymal cell manipulation that can lead to neurological improvement. To establish non-invasive ependymal cell targeting therapy, further studies are needed to advance our understanding of the precise molecular mechanism of the ependymal cell response for SCI, including their proliferation and differentiation.

## Materials and methods

### Animals

Adult C57BL/6J mice and IL-17ra floxed mice (Stock No: 031000) were purchased from Japan SLC, Inc. and Jackson Laboratory Laboratories, respectively. FoxJ1CreER^T2^::R26RtdTomato double transgenic mice were kindly provided by Dr. Jonas Frisen at Karolinska Institute. Mice were housed under specific pathogen-free conditions and had free access to water and food under a 12-h light/dark cycle with ambient temperature maintained at 24 °C. All experimental procedures were approved by the Institutional Animal Care Committee of Osaka University and complied with the guidelines for the care and use of laboratory animals at Osaka University.

### Tamoxifen administration

Tamoxifen (Sigma-Aldrich, St. Louis, MO, USA) was dissolved in corn oil (Sigma-Aldrich) at a concentration of 20 mg/ml by shaking overnight at 37 °C. Eight-week-old female mice received 100 µL of tamoxifen/corn oil through intraperitoneal administration once a day for 5 days. Mice were then used in the experiment after one day of clearance period.

### Spinal cord injury and animal care

Adult (8–10 weeks old) female mice were used for SCI experiments, because bladder expression is easier and the risk of complications is lower in female mice [45]. Mice were anesthetized through the intraperitoneal administration of a mixture of 0.3 mg/kg medetomidine hydrochloride (ZENOAQ, Fukushima, Japan), 4 mg/kg midazolam (Astellas Pharma, Tokyo, Japan), and 5 mg/kg butorphanol (Meiji Seika Pharma, Tokyo, Japan). Following dorsal laminectomy of T9–10, the mouse body was fixed, and the spinal cord was crushed with a force of 60 kilodyne (moderate/severe contusion [46]) using the Infinite Horizon Impactor (Precision Systems & Instrumentation, Fairfax Station, VA, USA). After surgery, the muscle and skin layers were sutured, and the mice were placed on a panel heater until they awoke. Mice that had only laminectomy and spinal cord exposure were included in the sham-operated group. The bladder was expressed by manual abdominal pressure every day until 14 days post-injury.

### Anti-IL-17A antibody treatment

Immediately after SCI in C57BL/6J mice, control mouse IgG (Sigma-Aldrich) or anti-IL-17A antibody (16-7173, Thermo Fisher Scientific, Waltham, MA, USA)-loaded osmotic minipumps (10 μg/100 μL solution, 0.5 µL/h, delivered for 7 days; 1007D, Alzet Corp, Cupertino, CA, USA) were implanted in each mouse. The minipump was placed subcutaneously on the back of the mouse, and the outlet of a silicone rubber tube connected to the minipump was placed at the injury site. The tube was sutured to the muscle at several points, and then the muscle and skin layers were sutured. Each sample was collected after behavioral analysis for 7 days and 8 weeks after SCI.

### RNA extraction and quantitative polymerase chain reaction

Using TRIzol reagent (Thermo Fisher Scientific), total RNA was extracted from the injured spinal cords of mice from the sham and SCI groups at each time point. The isolated total RNA was purified with the RNeasy Mini kit (QIAGEN N.V., Venlo, NLD), and the complementary DNA (cDNA) was synthesized with the PrimeScript^TM^II High Fidelity RT-PCR Kit (Takara Bio, Shiga, Japan). Expression of mRNA was detected using the QuantStudio 7 Flex Real-Time PCR System (Applied Biosystems, Waltham, MA, USA), and the PCR conditions were as follows: 95 °C for 20 s, followed by 40 cycles of 95 °C for 3 s and 60 °C for 30 s. Gene expression levels were calculated using the ΔΔCt method and then normalized against the mean of *GAPDH* expression levels. The primer sequences are described in Supplementary Table 1 [47].

The abbreviation of each gene is as follows: NGF, nerve growth factor; NT3, neurotrophin 3; NT4/5, neurotrophin 4/5; BDNF, brain-derived neurotrophic factor; GDNF, glial cell line derived neurotrophic factor; CNTF, ciliary neurotrophic factor; LIF, leukemia inhibitory factor; CNPase, 2′, 3′- cyclic nucleotide 3′- phosphodiesterase; bFGF, basic fibroblast growth factor; EGF, endothelial growth factor; VEGF-A, vascular endothelial growth factor A; GFAP, glial fibrillary acidic protein; IGF-1, insulin-like growth factor-1; PDGF-A, platelet-derived growth factor subunit A.

### Behavioral testing

To evaluate hind limb motor function after SCI, the Basso Mouse Scale (BMS) open-field locomotor test [48] and beam walk tests [49, 50] were employed. BMS scores were determined in accordance with the method of a previous study [48], and the data were obtained by averaging the scores of the right and left hindlimbs. The scores were recorded just before the surgery (Pre), at 1, 3, 7, and 14 days post-injury, and once weekly thereafter for a total of 8 weeks.

A subset of animals was assessed using a modified version of the beam walk test, which evaluates the severity of motor incoordination. The hindlimb movements of mice walking on a narrow wooden beam (width: 1.4 cm; length: 100 cm; height: 15 cm) were recorded, and the scores were determined with reference to a previous study [51]. Mice were pre-trained 2 days before surgery, and beam walk tests were performed just before the surgery (Pre), 14 days post-injury, and once weekly thereafter, for a total of 8 weeks. In each trial, the average scores of the right and left hind limbs were calculated and determined by the average of 3 trials per mouse. The grid walk test was also performed to assess hindlimb locomotion [52]. Mice were placed on a metal grid with 12 mm square hole and allowed to freely explore, and performance were recorded with video camera. When the hindpaw falls down, it is considered as a foot slip. The number of foot slip within 50 steps for each hindpaw was counted, and the mean percentage of foot slips was calculated. All behavioral analyses were performed under single-blind conditions.

### Tissue preparation and histological analysis

Mice were deeply anesthetized and transcardially perfused with ice-cold phosphate-buffered saline (PBS) followed by 4% paraformaldehyde in PBS. The spinal cords were harvested following perfusion, then immersed in the same fixatives overnight at 4 °C and 30% sucrose in PBS for 24 h at 4 °C. The tissues were embedded in Tissue-Tek O.C.T. Compound and frozen at −80 °C until use. The spinal cords were cut into 20- or 30-μm-thick transverse sections using a cryostat and mounted on MAS-coated glass slides (Matsunami, Osaka, Japan). After washing three times with PBS, the sections were blocked with PBS containing 0.1% Triton X-100 and 3% normal goat serum in PBS for 1 h at room temperature. Subsequently, the sections were incubated with primary antibody in blocking buffer overnight at 4 °C. The following antibodies were used as primary antibodies: rabbit anti-IL-17A antibody (1:100; ab79506, Abcom, Cambridge, England, UK), rat anti-CD45 antibody (1:500, 553076, BD Biosciences, San Jose, CA, USA), rat anti-CD68 antibody (1:1000; ab53444, Abcom), rabbit anti-Iba1 antibody (1:1000; 019-19741, Wako, Osaka, Japan), rabbit anti-IL17RAantibody (1:100; bs-2606R, Bioss Antibodies, Woburn, MA, USA), rabbit anti-Ki67 antibody (1:200; ab16667, Abcom), mouse anti-GFAP antibody (1:1000; G3893, Sigma- Aldrich), and rabbit anti-5-HT (serotonin) antibody (1:2000; 20080, Immunostar, Hudson, WI, USA). After washing with PBS, the sections were incubated with fluorescein-conjugated secondary antibodies in blocking solution for 2 h at room temperature in the dark. Alexa Fluor 488 goat anti-rabbit IgG, Alexa Fluor 488 goat anti-mouse IgG, Alexa Fluor 568 goat anti-mouse IgG, Alexa Fluor 568 goat anti-rat IgG, or Alexa Fluor 647 goat anti-rabbit IgG were used as secondary antibodies (1:500; A-27034, A-11001, A-11004, A-11077 and A-27040, respectively; Invitrogen, Carlsbad, CA, USA). The nuclei were stained with DAPI, 1 mg/ml; Dojindo Laboratories, Kumamoto, Japan). To visualize the biotin dextran amine (BDA)-labeled axons, the sections were incubated with Alexa Fluor 647 streptavidin (1:400; S-21374, Thermo Fisher Scientific) in blocking solution for 2 h at room temperature. The sections were then washed three times in PBS and mounted with a fluorescence mounting medium (Dako). Images were acquired using a fluorescence microscope (BX53, Olympus, Tokyo, Japan) and confocal laser-scanning microscopy (FV1200 and FV3000, Olympus). The quantification of fluorescence intensity in histological sections was expressed as threshold and measured using Image J software (NIH, Bethesda, MD, USA).

### Anterograde labeling of the CST

On day 28 or 42 after SCI, mice were anesthetized, and their heads were fixed on a stereotaxic frame, and the corresponding skull area was carefully exposed. To label the CST axons, we injected 10% BDA solution (10,000 MW, D1956, Thermo Fisher Scientific) into the hindlimb region of the cerebral motor cortex using a glass capillary attached to a microsyringe (coordinates from bregma: 0.5 mm posterior/0.5 mm lateral, 0.5 mm posterior/1.0 mm lateral, 1.0 mm posterior/0.5 mm lateral, 1.0 mm posterior/1.0 mm lateral, at a depth of 0.5 mm, 0.5 μL each). The mice were killed 2 weeks after BDA injection, and spinal cord tissues were harvested.

### Quantitative analysis

For analysis of the area of spared tissue, images of whole spinal cord cross-sections stained with GFAP were taken and analyzed using ImageJ software (NIH) [53, 54]. The spared tissue area was determined by manually enclosing the lesion border indicated by GFAP immunoreactivity, then calculating the total area for each section.

To quantify CST and 5-HT axons, we evaluated the axonal distribution by using transverse sections of the spinal cord [55, 56]. We obtained serial transverse sections (20 μm thickness) of the spinal cord and quantitatively analyzed the axonal distribution. We collected serial transverse spinal cord sections from the lesion epicenter to the 3 mm rostral and 3 mm caudal sides and quantified the densities of BDA- and 5-HT-positive axons [1, 53]. BDA-positive CST axons extending from the main CST to the gray matter were quantitatively analyzed by measuring the BDA-positive area. 5-HT-positive axons were evaluated by quantifying the 5-HT-positive area in the region of 750 µm square around the central canal (CC). Considering the tracing efficiency, data were normalized to the value obtained at a distance of 3 mm rostral to the lesion epicenter (normalized value is represented as the axon index) because the impact of contusion injury on the axon density at this site was minimal, and the data were quantitatively evaluated relative to the values at each site from the epicenter and presented as the percentage of fluorescence-positive areas. The quantification was calculated from three sections per site spaced 400 μm apart.

To evaluate the proliferation ability of ependymal cells, the number of Ki67-positive cells co-labeled with DAPI within the CC ependymal zone was counted. Forty serial coronal sections (30 μm thickness) were continuously taken from the rostral side of the lesion center, and the number of Ki67-positive cells per section was quantified and averaged. FoxJ1CreER^T2^::R26R tdTomato mice [6] were used to evaluate the proliferation ability of ependymal cell progeny following SCI. Sequential spinal cord sections (30 μm thickness) 3 mm rostral (R) to 3 mm caudal (C) from the lesion epicenter were collected and the tdTomato-positive area was quantified. The quantification was performed from two sections per site, spaced 300 μm apart. The proportion (area ratio) of GFAP and Iba1 staining (GFAP-positive area/total area of ROI, and Iba1-positive area/total area of ROI) was calculated using Image J software (Bethesda, MD, USA). Three axial sections for each spinal segment (0.8 mm rostral to the lesion epicenter, lesion epicenter, and 0.8 mm caudal to lesion epicenter) were quantified.

### Statistical analysis

All data are presented as mean ± standard error of the mean (SEM), and *P* < 0.05 was considered to represent a significant difference. The unpaired two-sided Student’s *t* test and Mann–Whitney *U* test were used for comparisons between two groups, and one-way or two-way analysis of variance (ANOVA) with post hoc Tukey-Kramer tests or Bonferroni tests were used for multiple groups. A repeated-measures two-way ANOVA with post-hoc Bonferroni tests was used for assessing the data of the behavioral test. Details of each statistical test are indicated in the figure legends. Power calculations to predetermine sample sizes were not performed, and the sample size of each group was determined based on previous publications [1, 23, 53]. No randomization was used. Statistical analyses were conducted using GraphPad Prism 8 software (GraphPad Software, San Diego, CA, USA).

## Supplementary information

Supplementary Figure 1

Supplementary Figure 2

Supplementary Figure 3

Supplementary Table 1

## Data Availability

All data relevant for the study are shown in the manuscript or the supplementary materials. The data that support the findings of this report are available from the corresponding author upon reasonable request.

## References

[1] Anderson MA, Burda JE, Ren Y, Ao Y, O’Shea TM, Kawaguchi R (2016). Astrocyte scar formation aids central nervous system axon regeneration. Nature..

[2] Barnabe-Heider F, Goritz C, Sabelstrom H, Takebayashi H, Pfrieger FW, Meletis K (2010). Origin of new glial cells in intact and injured adult spinal cord. Cell Stem Cell.

[3] Brambilla R, Bracchi-Ricard V, Hu WH, Frydel B, Bramwell A, Karmally S (2005). Inhibition of astroglial nuclear factor kappaB reduces inflammation and improves functional recovery after spinal cord injury. J Exp Med.

[4] Meletis K, Barnabe-Heider F, Carlen M, Evergren E, Tomilin N, Shupliakov O (2008). Spinal cord injury reveals multilineage differentiation of ependymal cells. PLoS Biol.

[5] Menet V, Prieto M, Privat A, Giménez y Ribotta M (2003). Axonal plasticity and functional recovery after spinal cord injury in mice deficient in both glial fibrillary acidic protein and vimentin genes. Proc Natl Acad Sci USA.

[6] Sabelström H, Stenudd M, Réu P, Dias DO, Elfineh M, Zdunek S (2013). Resident neural stem cells restrict tissue damage and neuronal loss after spinal cord injury in mice. Science.

[7] Stenudd M, Sabelström H, Frisén J (2015). Role of endogenous neural stem cells in spinal cord injury and repair. JAMA Neurol.

[8] Thuret S, Moon LD, Gage FH (2006). Therapeutic interventions after spinal cord injury. Nat Rev Neurosci.

[9] Yiu G, He Z (2006). Glial inhibition of CNS axon regeneration. Nat Rev Neurosci.

[10] Rolls A, Shechter R, Schwartz M (2009). The bright side of the glial scar in CNS repair. Nat Rev Neurosci.

[11] Afshari FT, Kappagantula S, Fawcett JW (2009). Extrinsic and intrinsic factors controlling axonal regeneration after spinal cord injury. Expert Rev Mol Med.

[12] Busch SA, Silver J (2007). The role of extracellular matrix in CNS regeneration. Curr Opin Neurobiol.

[13] McKeon RJ, Schreiber RC, Rudge JS, Silver J (1991). Reduction of neurite outgrowth in a model of glial scarring following CNS injury is correlated with the expression of inhibitory molecules on reactive astrocytes. J Neurosci.

[14] Yuan YM, He C (2013). The glial scar in spinal cord injury and repair. Neurosci Bull.

[15] Del Bigio MR (2010). Ependymal cells: biology and pathology. Acta Neuropathol.

[16] Becker CG, Becker T, Hugnot JP (2018). The spinal ependymal zone as a source of endogenous repair cells across vertebrates. Prog Neurobiol.

[17] Lacroix S, Hamilton LK, Vaugeois A, Beaudoin S, Breault-Dugas C, Pineau I (2014). Central canal ependymal cells proliferate extensively in response to traumatic spinal cord injury but not demyelinating lesions. PLoS ONE.

[18] Iwakura Y, Ishigame H, Saijo S, Nakae S (2011). Functional specialization of interleukin-17 family members. Immunity..

[19] Komiyama Y, Nakae S, Matsuki T, Nambu A, Ishigame H, Kakuta S (2006). IL-17 plays an important role in the development of experimental autoimmune encephalomyelitis. J Immunol.

[20] Li Z, Li K, Zhu L, Kan Q, Yan Y, Kumar P (2013). Inhibitory effect of IL-17 on neural stem cell proliferation and neural cell differentiation. BMC Immunol.

[21] Liu Q, Xin W, He P, Turner D, Yin J, Gan Y (2014). Interleukin-17 inhibits adult hippocampal neurogenesis. Sci Rep.

[22] Zong S, Zeng G, Fang Y, Peng J, Tao Y, Li K (2014). The role of IL-17 promotes spinal cord neuroinflammation via activation of the transcription factor STAT3 after spinal cord injury in the rat. Mediators Inflamm.

[23] Hill F, Kim CF, Gorrie CA, Moalem-Taylor G (2011). Interleukin-17 deficiency improves locomotor recovery and tissue sparing after spinal cord contusion injury in mice. Neurosci Lett.

[24] Perrin FE, Noristani HN (2019). Serotonergic mechanisms in spinal cord injury. Exp Neurol.

[25] Tuszynski MH, Steward O (2012). Concepts and methods for the study of axonal regeneration in the CNS. Neuron.

[26] Lee CM, Zhou L, Liu J, Shi J, Geng Y, Liu M (2020). Single-cell RNA-seq analysis revealed long-lasting adverse effects of tamoxifen on neurogenesis in prenatal and adult brains. Proc Natl Acad Sci USA.

[27] Harvey AR, Lovett SJ, Majda BT, Yoon JH, Wheeler LP, Hodgetts SI (2015). Neurotrophic factors for spinal cord repair: Which, where, how and when to apply, and for what period of time?. Brain Res.

[28] Hawryluk GW, Mothe AJ, Chamankhah M, Wang J, Tator C, Fehlings MG (2012). In vitro characterization of trophic factor expression in neural precursor cells. Stem Cells Dev.

[29] Choi GB, Yim YS, Wong H, Kim S, Kim H, Kim SV (2016). The maternal interleukin-17a pathway in mice promotes autism-like phenotypes in offspring. Science..

[30] Reed MD, Yim YS, Wimmer RD, Kim H, Ryu C, Welch GM (2020). IL-17a promotes sociability in mouse models of neurodevelopmental disorders. Nature..

[31] Shichita T, Sugiyama Y, Ooboshi H, Sugimori H, Nakagawa R, Takada I (2009). Pivotal role of cerebral interleukin-17-producing gammadeltaT cells in the delayed phase of ischemic brain injury. Nat Med.

[32] Li GZ, Zhong D, Yang LM, Sun B, Zhong ZH, Yin YH (2005). Expression of interleukin-17 in ischemic brain tissue. Scand J Immunol.

[33] Li T, Zhang YM, Han D, Hua R, Guo BN, Hu SQ (2017). Involvement of IL-17 in secondary brain injury after a traumatic brain injury in rats. Neuromolecular Med.

[34] Gao L, Li PP, Shao TY, Mao X, Qi H, Wu BS (2020). Neurotoxic role of interleukin-17 in neural stem cell differentiation after intracerebral hemorrhage. Neural Regen. Res.

[35] Li X, Floriddia EM, Toskas K, Fernandes KJL, Guerout N, Barnabe-Heider F (2016). Regenerative potential of ependymal cells for spinal cord injuries over time. EBioMedicine.

[36] Paniagua-Torija B, Norenberg M, Arevalo-Martin A, Carballosa-Gautam MM, Campos-Martin Y, Molina-Holgado E (2018). Cells in the adult human spinal cord ependymal region do not proliferate after injury. J Pathol.

[37] Ren Y, Ao Y, O’Shea TM, Burda JE, Bernstein AM, Brumm AJ (2017). Ependymal cell contribution to scar formation after spinal cord injury is minimal, local and dependent on direct ependymal injury. Sci Rep.

[38] Daglas M, Draxler DF, Ho H, McCutcheon F, Galle A, Au AE (2019). Activated CD8(+) T cells cause long-term neurological impairment after traumatic brain injury in mice. Cell Rep.

[39] Zhang Q, Liao Y, Liu Z, Dai Y, Li Y, Li Y (2021). Interleukin-17 and ischaemic stroke. Immunology.

[40] Llorens-Bobadilla E, Chell JM, Le Merre P, Wu Y, Zamboni M, Bergenstrahle J, et al. A latent lineage potential in resident neural stem cells enables spinal cord repair. Science. 2020;370:eabb8795. 10.1126/science.abb8795 33004487

[41] Griffin JM, Bradke F (2020). Therapeutic repair for spinal cord injury: combinatory approaches to address a multifaceted problem. EMBO Mol Med.

[42] Tang XQ, Tanelian DL, Smith GM (2004). Semaphorin3A inhibits nerve growth factor-induced sprouting of nociceptive afferents in adult rat spinal cord. J Neurosci.

[43] Fischer I, Dulin JN, Lane MA (2020). Transplanting neural progenitor cells to restore connectivity after spinal cord injury. Nat Rev Neurosci.

[44] Ruff CA, Wilcox JT, Fehlings MG (2012). Cell-based transplantation strategies to promote plasticity following spinal cord injury. Exp Neurol.

[45] Lilley E, Andrews MR, Bradbury EJ, Elliott H, Hawkins P, Ichiyama RM (2020). Refining rodent models of spinal cord injury. Exp Neurol.

[46] Nishi RA, Liu H, Chu Y, Hamamura M, Su MY, Nalcioglu O (2007). Behavioral, histological, and ex vivo magnetic resonance imaging assessment of graded contusion spinal cord injury in mice. J Neurotrauma.

[47] Hawryluk GW, Mothe A, Wang J, Wang S, Tator C, Fehlings MG (2012). An in vivo characterization of trophic factor production following neural precursor cell or bone marrow stromal cell transplantation for spinal cord injury. Stem Cells Dev.

[48] Basso DM, Fisher LC, Anderson AJ, Jakeman LB, McTigue DM, Popovich PG (2006). Basso Mouse Scale for locomotion detects differences in recovery after spinal cord injury in five common mouse strains. J Neurotrauma.

[49] Hill RL, Zhang YP, Burke DA, Devries WH, Zhang Y, Magnuson DS (2009). Anatomical and functional outcomes following a precise, graded, dorsal laceration spinal cord injury in C57BL/6 mice. J Neurotrauma.

[50] Metz GA, Merkler D, Dietz V, Schwab ME, Fouad K (2000). Efficient testing of motor function in spinal cord injured rats. Brain Res.

[51] Nakamura Y, Fujita Y, Ueno M, Takai T, Yamashita T (2011). Paired immunoglobulin-like receptor B knockout does not enhance axonal regeneration or locomotor recovery after spinal cord injury. J Biol Chem.

[52] Zhang S, Fujita Y, Matsuzaki R, Yamashita T (2018). Class I histone deacetylase (HDAC) inhibitor CI-994 promotes functional recovery following spinal cord injury. Cell Death Dis.

[53] Ishii H, Jin X, Ueno M, Tanabe S, Kubo T, Serada S (2012). Adoptive transfer of Th1-conditioned lymphocytes promotes axonal remodeling and functional recovery after spinal cord injury. Cell Death Dis.

[54] Duncan GJ, Manesh SB, Hilton BJ, Assinck P, Liu J, Moulson A (2018). Locomotor recovery following contusive spinal cord injury does not require oligodendrocyte remyelination. Nat Commun.

[55] Steward O, Zheng B, Tessier-Lavigne M (2003). False resurrections: distinguishing regenerated from spared axons in the injured central nervous system. J Comp Neurol.

[56] Ito S, Nagoshi N, Tsuji O, Shibata S, Shinozaki M, Kawabata S, et al. LOTUS inhibits neuronal apoptosis and promotes tract regeneration in contusive spinal cord injury model mice. eNeuro. 2018;5:ENEURO.0303-18.2018. 10.1523/ENEURO.0303-18.2018 PMC6294604 30560203

