# Supplementary figures and images for: Interleukin-17A regulates ependymal cell proliferation and functional recovery after spinal cord injury in mice
Source: Cell Death Dis. 2021 Aug 3;12(8):766. doi: 10.1038/s41419-021-04064-1 (PMC8333070; doi:10.1038/s41419-021-04064-1)

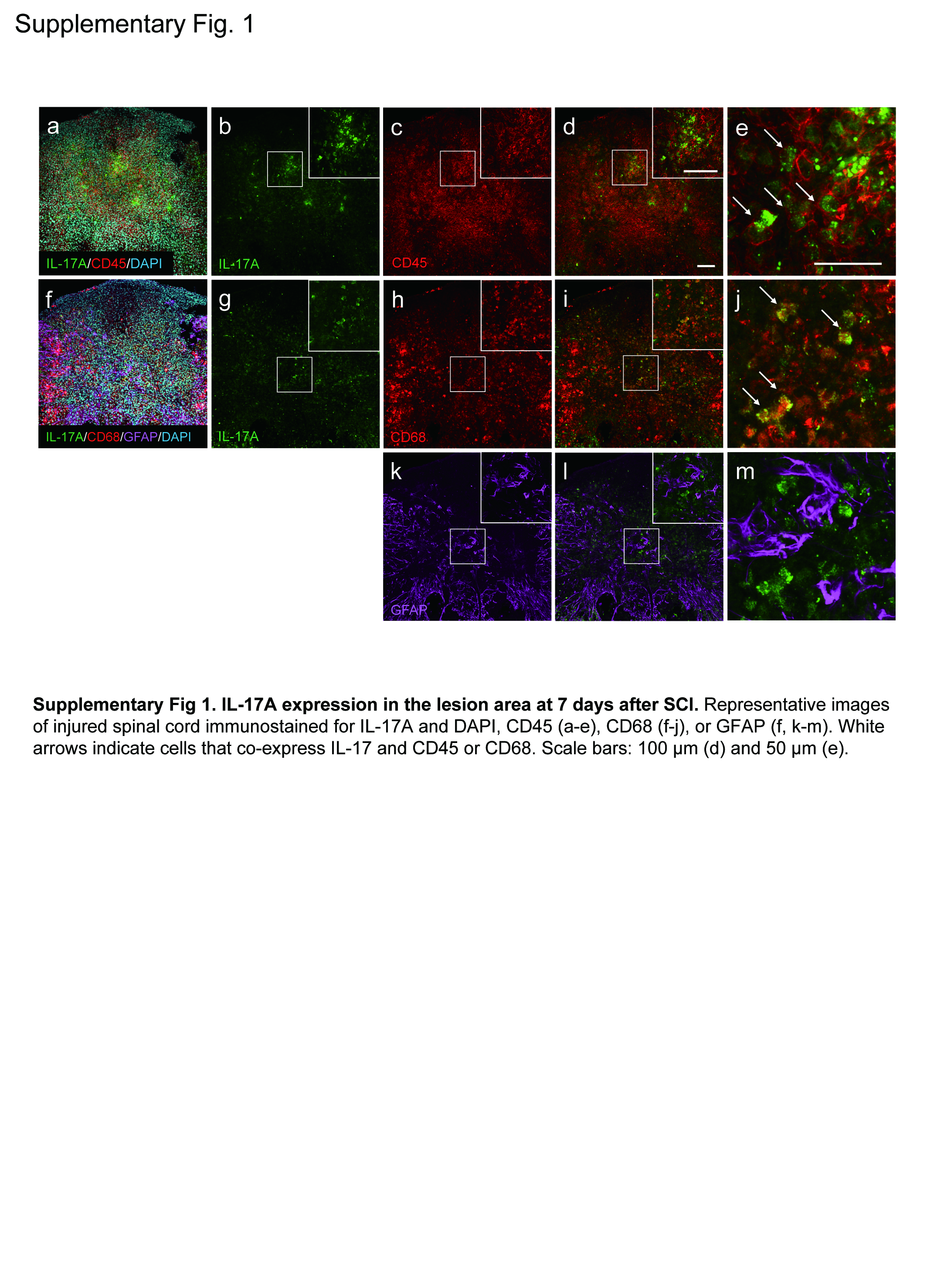

Supplement: Supplementary file 1 — Supplementary Figure 1 [file 41419_2021_4064_MOESM1_ESM.tif]

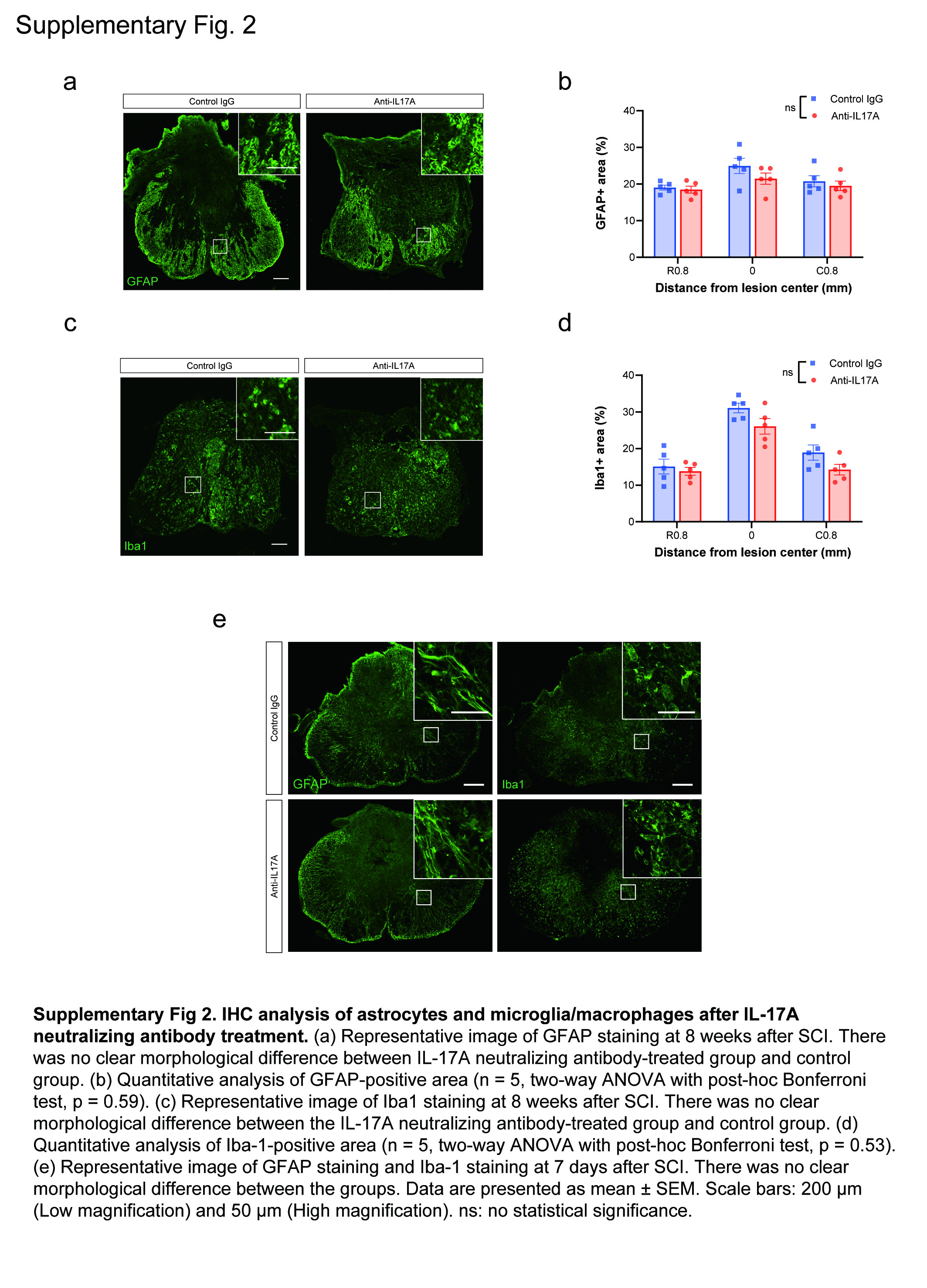

Supplement: Supplementary file 2 — Supplementary Figure 2 [file 41419_2021_4064_MOESM2_ESM.tif]

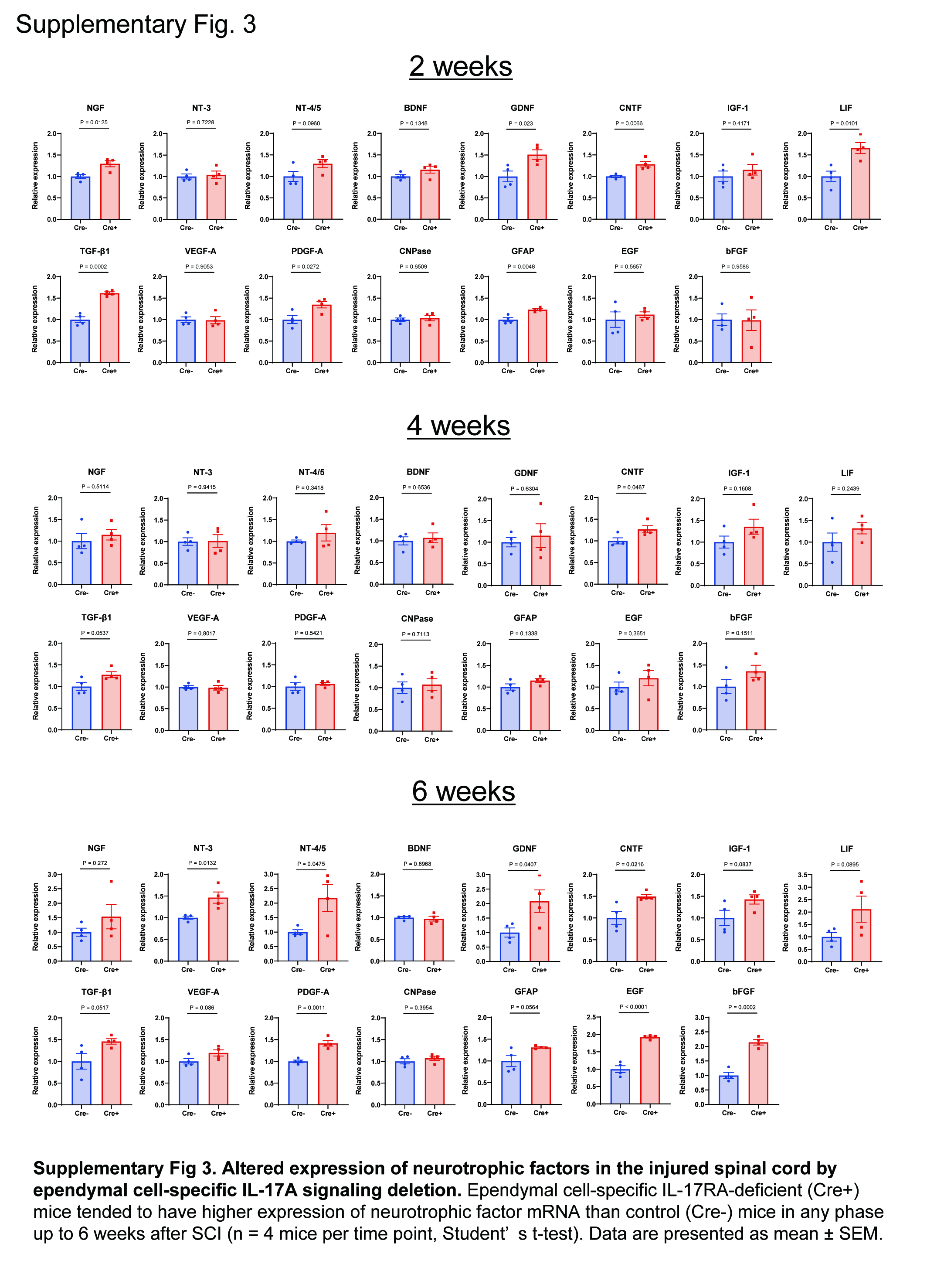

Supplement: Supplementary file 3 — Supplementary Figure 3 [file 41419_2021_4064_MOESM3_ESM.tif]

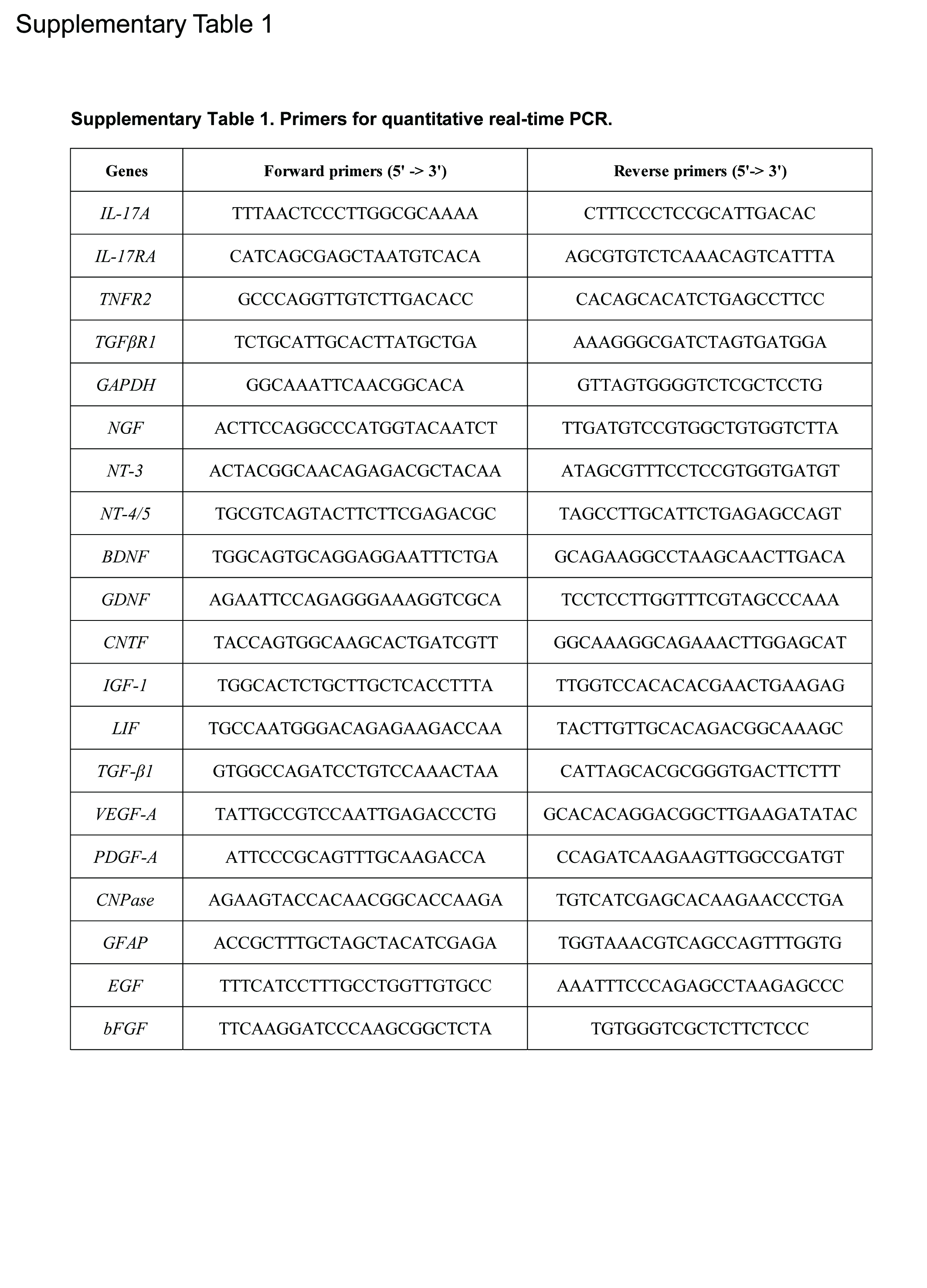

Supplement: Supplementary file 4 — Supplementary Table 1 [file 41419_2021_4064_MOESM4_ESM.tif]
